# Evaluating optimal utilisation of technology in type 1 diabetes mellitus from a clinical and health economic perspective: protocol for a systematic review

**DOI:** 10.1186/s13643-018-0706-9

**Published:** 2018-03-12

**Authors:** Anthony Pease, Clement Lo, Arul Earnest, Danny Liew, Sophia Zoungas

**Affiliations:** 1 Division of Metabolism, Ageing and Genomics, Department of Epidemiology and Preventive Medicine, School of Public Health and Preventive Medicine, Monash University, 5th Floor, 99 Commercial Road, Melbourne, VIC 3004 Australia; 2 Monash Centre for Health Research and Implementation, School of Public Health and Preventive Medicine, Monash University in partnership with Monash Health, Clayton, VIC 3168 Australia; 3 Division of Clinical Epidemiology, Centre of Cardiovascular Research and Education in Therapeutics, School of Public Health and Preventive Medicine, Monash University, Melbourne, VIC 3004 Australia

**Keywords:** Type 1 diabetes mellitus, Technology, Cost-effectiveness, Clinical effectiveness

## Abstract

**Background:**

Technology has been implemented since the 1970s with the hope of improving glycaemic control and reducing the burden of complications for those living with type 1 diabetes. A clinical and cost-effectiveness comparison of all available technologies including continuous subcutaneous insulin infusion (CSII), continuous glucose monitors (CGMs), sensor-augmented pump therapy (including either low-glucose suspend or predictive low-glucose suspend), hybrid closed-loop systems, closed-loop (single-hormone or dual-hormone) systems, flash glucose monitoring (FGM), insulin bolus calculators, and ‘smart-device’ applications is currently lacking. This systematic review, network meta-analysis, and narrative synthesis aims to summarise available evidence regarding the clinical and cost-effectiveness of available technologies in the management of patients with type 1 diabetes.

**Methods:**

Relevant studies will be searched using a comprehensive strategy through MEDLINE, MEDLINE in-process and other non-indexed citations, EMBASE, PubMed, all evidenced-based medicine reviews, EconLit, Cost-effectiveness Analysis Registry, Research Papers in Economics, Web of Science, PsycInfo, CINAHL, and PROSPERO for randomised controlled trials and economic evaluations. The search strategy will assess if there are combinations of currently available technologies that are superior to each other or to insulin injections and capillary blood glucose testing with regard to glycaemic control, morbidity/mortality, quality of life, and cost-effectiveness. Two reviewers will screen all articles for eligibility and then independently evaluate risk of bias, complete quality assessment, and extract data for included studies. Network meta-analyses will be performed where there is sufficient homogenous clinical data. Narrative synthesis will be performed for heterogeneous clinical data that cannot be pooled for network meta-analysis with critical appraisal of economic evaluations.

**Discussion:**

This systematic review protocol utilises rigorous methodology and pre-determined eligibility criteria to provide a uniquely comprehensive search for a broad spectrum of clinical and economic outcomes in comparing multiple currently available technologies for managing type 1 diabetes. Evidence on which technologies may be most appropriate for particular patient groups will be examined as well as the economic justification for funding of different technologies.

**Systematic review registration:**

PROSPERO (CRD42017077221)

**Electronic supplementary material:**

The online version of this article (10.1186/s13643-018-0706-9) contains supplementary material, which is available to authorized users.

## Background

Type 1 diabetes is an autoimmune condition primarily affecting pancreatic beta islet cells, leading to absolute insulin deficiency and hyperglycaemia [1]. Over 120,000 Australians are currently living with type 1 diabetes and are dependent on injected insulin titrated to food, exercise, stress, and illness [2–4]. The potential consequences of sub-optimal glycaemic control are multi-systemic in nature, leading to substantial morbidity and markedly increased mortality rates [1–3]. This exerts a significant impact on those effected, their carers, and healthcare costs. Annual nationwide diabetes-related total costs are estimated to be between $430 and $570 million (AUD) [5, 6].

Technologies have been implemented as early as the 1970s in an effort to control blood glucose levels (BGLs). Since then, there has been rapid development and production of devices and applications to facilitate more accurate insulin delivery and improve the precision and ease of measuring blood glucose levels. However, new technologies can be expensive and often need to be privately funded [7]. The average costs to consumers of available technologies are not clear, and estimates vary significantly depending on the extent of use. For example, the use of continuous glucose monitor systems may result in annual costs of ~$5000 (AUD) for the user [8]. Further, insulin pumps may cost ~$9500 (AUD) and are typically replaced every 4–6 years [8, 9].

A broad clinical and cost comparison of all available technologies in type 1 diabetes management including continuous subcutaneous insulin infusion (CSII), continuous glucose monitors (CGMs), sensor-augmented pump (SAP) therapy (including either low-glucose suspend or predictive low-glucose suspend), hybrid closed-loop systems, closed-loop (single-hormone or dual hormone) systems, flash glucose monitoring (FGM), insulin bolus calculators, and ‘smart’ device applications is currently lacking. Existing reviews have considered some forms of CSII with or without CGM technology, but there is no consensus on cost-effectiveness or in whom these devices should be targeted or avoided. Among those advocating technology as cost-effective, the justification has often been based on a sustained 0.5% reduction in glycated haemoglobin (HbA1c) and thus a reduced risk of end-organ complications [6, 10]. Reduction in severe hypoglycaemia is another outcome with potential for substantial financial impact [6, 10]. To date, randomised controlled trials (RCTs) have reported variable efficacy in reaching glycaemic targets, and yet predictive economic models typically focus on effects of this outcome alone [11, 12].

Economic evaluation of technology in the management of type 1 diabetes ranges from trial data with basic costing, through complex models with computer simulations. A minority of existing models consider type 1 diabetes primarily, and while various proprietary models are available, these may come at an expense. Cost-effectiveness data in the Australian healthcare setting is a particular deficit in light of new initiatives to fund some technologies in the paediatric population [13]. The rapid pace of technology development has also left many reviews outdated. This systematic review and network meta-analysis aims to provide a comprehensive clinical and economic comparison of currently available technologies. The primary target audience will be endocrinologists, other clinicians caring for people with type 1 diabetes, those with an interest in health technology, patients with and carers of those with type 1 diabetes, and policy makers in the healthcare setting.

### Objectives of the systematic review

The objective of this study is to determine if any currently available technology utilised in managing adults with type 1 diabetes is superior to other technologies or to insulin injections and capillary blood glucose testing for achieving improved glycaemic control, lower risk of complications, superior quality of life and more favourable cost-effectiveness.

## Methods

### Systematic review design

A comprehensive search strategy was developed and translated for each database to incorporate relevant participants, interventions, comparators, and outcomes (PICO; see Additional file 1). Reference lists of included studies will also be screened manually. Systematic review and subsequent network meta-analysis will be conducted following methods outlined in the Cochrane Handbook for Systematic Reviews of Interventions guidelines [14] and conforming to the reporting guidelines of the Preferred Reporting Items for Systematic Reviews and Meta-Analysis for Protocols (PRISMA-P) statement [15, 16] and the PRISMA extension statement for systematic reviews incorporating network meta-analyses of healthcare interventions [17]. A populated PRISMA-P checklist is provided (Additional file 2) [16]. Systematic review of economic outcomes will also conform to the Consolidated Health Economic Evaluation Reporting Standards (CHEERS) [18].

### Population/participants

Community dwelling, non-pregnant, adult patients (18 years of age or over) from any country, with type 1 diabetes who are being managed in an outpatient setting, will be included. Study populations involving additional demographics may be included only if results are stratified by our inclusion criteria, or this information is provided on request.

### Interventions

Broadly, methods of insulin delivery, blood glucose monitoring, and advising insulin doses will be considered. These methods include:

- Multiple daily injections (MDI): (basal bolus)
- Self-monitoring of blood glucose via capillary testing (SMBG)
- Continuous subcutaneous insulin infusion (CSII) systems, CSII with a low-glucose suspend feature, CSII with a predictive low-glucose suspend feature, hybrid closed-loop CSII systems, closed-loop (insulin only or insulin and glucagon) CSII systems
- Continuous glucose monitors (CGM)
- Flash glucose monitors (FGM)
- Insulin bolus calculators
- Smart device applications

Other forms of technology not listed in the protocol will be excluded. No date limitations were used in the search strategy.

### Comparator

For the purposes of network meta-analysis, the technology-based interventions will be compared with each other or combinations of technology types either directly or indirectly depending on available evidence. All technology-based interventions will also be compared to capillary blood glucose testing with basal bolus insulin injections. Narrative synthesis and review will only consider direct comparisons.

### Outcomes

#### Primary outcomes

Note that only clinical outcomes will be included in the network meta-analysis.

- HbA1c results:
  - ◦ Achieving targets:
    - ■ < 6.5% (48 mmol/mol)
    - ■ ≤ 7.0% (53 mmo/mol)
    - ■ ≤ 8.0% (64 mmol/mol)
  - ◦ Change from baseline HbA1c % (mmol/mol)
- Hypoglycaemia:
  - ◦ Frequency and total number of events per unit of time
  - ◦ Severity category if provided by clinical trial:
    - ■ Level 1: 3.0 mmol/L (54 mg/dL) ≤ BGL < 3.9 mmol/L (70 mg/dL)
    - ■ Level 2: BGL < 3.0 mmol/L (54 mg/dL).
    - ■ Level 3: altered mental and/or physical status requiring third party assistance
  - ◦ Emergency services and/or hospital presentation or admission if provided
- Costs for economic evaluations:
  - ◦ Direct and indirect if provided
  - ◦ Incremental cost-effectiveness ratio (ICER)/quality-adjusted life-years (QALYs)

#### Secondary outcomes

- Hyperglycaemia:
  - ◦ Frequency and total number of events per unit of time
  - ◦ Severity category if provided:
    - ■ Elevated: 10.0 mmol/L (180 mg/dL) < BGL ≤ 13.9 mmol/L (250 mg/dL)
    - ■ Very elevated: BGL > 13.9 mmol/L (250 mg/dL)
  - ◦ Emergency services and/or hospital presentation or admission if provided
- Measured average blood glucose level (BGL)
- Estimated average blood glucose level
- Time in target/above or below target
  - ◦ Percentage of BGLs in the range of 3.9 mmol/L (70 mg/dL)–10.0 mmol/L (180 mg/dL) per unit of time
- Average fasting and post-prandial glucose levels in mmol/L (mg/dL)
- Average total daily dose of insulin being administered
  - ◦ Insulin sensitivity factors (and how calculated)
  - ◦ Insulin/carbohydrate ratios (and how calculated)
- Number of episodes of diabetic ketoacidosis (DKA) per unit of time of follow-up
  - ◦ Number of episodes of ketosis without DKA per unit of time
    - ■ Blood ketones: positive, or ≥ 0.6 mmol/L
    - ■ Urine ketones: positive, or ‘moderate to large’
  - ◦ Number of ketone tests per unit of time
- CSII and/or CGM discontinuation apart from trial protocol
- Measure of health-related quality of life using a validated tool, if provided
- Measure of health literacy/self-efficacy, if provided
- Engagement with health services, if provided
  - ◦ Number of clinic visits per unit of time
  - ◦ Number of clinics where the patient failed to attend per number of clinic visits offered per unit of time
- Complications of diabetes:
  - ◦ Diabetic retinopathy, peripheral neuropathy, nephropathy/end-stage kidney disease (ESKD), ischaemic heart disease (IHD), cerebrovascular accident (CVA), peripheral vascular disease (PVD), and autonomic neuropathy
- Mortality
- Measure of morbidity/Charlson Comorbidity Index (CMI) if detail provided
- Patient acceptability of testing method and method of insulin delivery
- Anxiety about hypoglycaemia or hyperglycaemia
- Adverse events from testing or treatment
  - ◦ False results: significant disagreement when comparing technology-based measurement system with gold standard (if provided)
  - ◦ Treatment errors: if measurement system or delivery system fails to perform its function (i.e. unintentionally stops measuring blood glucose or unintentionally stops delivering insulin/glucagon)

### Setting

RCTs and economic evaluations will be included from any region.

### Study design

For clinical outcomes, only randomised controlled trials of 6-week duration or longer will be included. The search for economic outcomes will include both RCT- and model-based evaluations. Cost-utility, cost-benefit, cost-effectiveness, and budget impact analyses may be considered.

### Search methods

The following clinical and economic electronic databases will be utilised to identify relevant literature using a systematic search strategy (Additional file 1):

- MEDLINE
- MEDLINE in-process and other non-indexed citations
- EMBASE
- PubMed
- All evidence-based medicine reviews, incorporating:
  - ◦ Cochrane Database of Systematic Reviews (via Wiley Online Library)
  - ◦ American College of Physicians Journal Club
  - ◦ Database of Abstracts of Reviews of Effects (via Wiley Online Library)
  - ◦ Cochrane Central Register of Controlled Trials (via Wiley Online Library)
  - ◦ Cochrane Methodology Register (via Wiley Online Library)
  - ◦ Health Technology Assessment (via Wiley Online Library)
  - ◦ NHS Economic Evaluation Database
- EconLit (EBSCOHost)
- Cost-effectiveness Analysis Registry (www.cearegistry.org)
- Research Papers in Economics (http://repec.org/)
- Web of Science
- PsycInfo
- CINAHL
- PROSPERO (www.crd.york.ac.uk/prospero/)

To identify ongoing trials, the International Clinical Trials Registry Platform Search Portal (http://apps.who.int/trialsearch/) will be used. This provides access to a central database containing the trial registration datasets provided by 16 different international registries. EndNote X8 (©2017 Clarivate Analytics) and Covidence (©2017 Covidence) will be utilised to manage articles throughout initial article screening, assessment of quality, and data extraction.

### Inclusion of studies

Two reviewers (AP and CL) will read titles, abstracts, and keywords of records retrieved by the search strategy for initial screening. Disagreement will be resolved by discussion, then deferral to a third reviewer (SZ). Full-text articles will be reviewed if the available information indicates that the study meets our inclusion criteria or if there is doubt based on the title and/or abstract. Similarly for full-text review, disagreements between AP and CL will be resolved by consensus or a third reviewer (SZ). Level of agreement on study eligibility will be tested using the kappa statistic and 95% confidence interval. A list of studies excluded at the full-text stage of screening will be provided along with the rationale for exclusion.

### Assessment of methodological quality

Methodological quality of the included studies will be assessed by two reviewers using published expert recommended tools for randomised controlled trials [19] as well as for trial- and model-based economic evaluations [20–22]. Any disagreement between the two reviewers not resolved by discussion may be referred to the third reviewer (SZ).

### Quality of evidence

Outcomes of interest will be reviewed utilising recommendations by the ‘Grading of Recommendations, Assessment, Development and Evaluation’ (GRADE) working group [23]. The direction and magnitude of effect estimate will be assessed. Quality of evidence will consider risk of bias, inconsistency, indirectness, imprecision, and publication bias. Overall quality will be classified as high, moderate, low, and very low. Randomised controlled trials will start with high-quality rating with each consideration being downgraded by 1 or 2 points as necessary. This may incorporate trial-based economic evaluations. The final quality score will be interpreted within the GRADE working group’s framework [23]. Model-based economic evaluations may undergo complementary assessment with the National Institute for Health and Care Excellence in United Kingdom (NICE) checklist [24, 25].

### Data extraction

A specifically developed data extraction form will be utilised for outcomes relevant to the review selection criteria. Information will be collected including general details (title, authors, reference/source, country, year of publication, setting, study design, type of economic evaluation with analytic approach, study perspective, duration of intervention, duration of follow-up, sources of funding, competing interests), eligibility criteria for study protocol, participants (number randomised, number in groups at follow-up, age, sex, race/ethnicity, selection criteria, recruitment method, withdrawals/losses to follow-up, subgroups, baseline imbalances, co-morbidities), interventions with comparisons (description, timing, delivery, providers, co-interventions, resource requirements, compliance, integrity of delivery), results (resource use, costs (type, category, method for calculation, disaggregated and aggregated), valuation methods, time horizon, discount rate for costs/effects, inflation rate, reference year, point estimates with measure of variability, frequency counts for dichotomous variables, intention-to-treat analysis or per protocol, imputation, power and sample size calculations, statistical methods/appropriateness, reanalysis required/possible, incremental cost-effectiveness ratios, uncertainty and sensitivity analyses), and any other relevant validity results. Information will also be collected for economic models relating to the external validation, structure, assumptions, and input data [26]. Missing data will be obtained from the authors wherever possible.

Where available, costs and ICERs will be expressed as published in the currency of the publisher (assumed to be the year preceding publication unless cost years are stated) and converted to 2018 Australian dollars (AUD) and US dollars (USD) using relevant exchange rates [27] and inflation rates [28].

### Data analysis and synthesis of evidence

Data will be presented in summary tables. Clinical results will be summarised statistically in network meta-analyses if data are available and sufficiently homogenous. Clinical homogeneity will be satisfied when participants, interventions, outcome measures and timing of outcome measurement are considered to be similar. Stata™ for Windows version 14.0 (StataCorp LP, College Station, TX, USA) and the Review Manager version 5.3.5 (Copenhagen: The Nordic Cochrane Centre, The Cochrane Collaboration, 2014) software will be used for direct and indirect network meta-analysis. Reporting will follow the Preferred Reporting Items for Systematic Reviews and Meta-Analyses (PRISMA) extension statement for systematic reviews incorporating network meta-analyses of healthcare interventions [17]. Economic evaluations will be ranked on a league table with results converted to 2018 AUD using relevant exchange rates [27] and inflation rates [28]. Disaggregated results may be provided and cost-effectiveness planes utilised to display a summary of economic data. Assessment of transferability of economic evaluations may also be performed using ‘a checklist to frame health technology assessments for resource allocation decisions’ [29].

For clinical outcomes, statistical homogeneity will be assessed using the *I*^2^ test where *I*^2^ values over 50% indicate moderate to high heterogeneity [30]. Statistical significance will be set at *P* < 0.05. Results of trials with moderate to high heterogeneity will be pooled and analysed using a random effects model, and trials with low heterogeneity will be analysed using a fixed effects model. Network meta-analysis results will be expressed as relative risks (RR) with 95% confidence intervals (CI) for dichotomous outcomes and mean differences (MD) with 95% CI for continuous outcomes.

Indirect treatment comparisons will be utilised if adequate data is presented in the absence of direct comparisons. Assumptions of homogeneity, similarity, and consistency will be applied and combined with direct comparisons, consistent with International Society for Pharmacoeconomics and Outcomes Research (ISPOR) taskforce recommendations [22, 26, 31, 32]. Only studies that provide equivalent outcome measures at equal duration of follow-up will be compared with each other. Depending on the data available, further subgroup analysis may be performed as specified in the ‘Subgroup analysis’ section of this protocol.

Narrative synthesis will be performed for heterogeneous clinical data that cannot be pooled for network meta-analysis. Methodological quality, quality of evidence, and data extraction will follow the same methods as above (see ‘Assessment of methodological quality’, ‘Quality of evidence’, and ‘Data extraction’ sections). Studies will be grouped by the type of interventions and outcomes considered. Results will be tabulated and discussed with forest plots for primary outcomes. Idea webbing may be performed if relationships are not addressed by the network meta-analysis. [33, 34]

### Subgroup analysis

As part of network meta-analysis and narrative synthesis, subgroup analysis will be conducted if baseline data is available regarding:

- Failure to reach target HbA1c despite intensive therapy
- Poor HbA1c (> 9.0%) despite intensive therapy
- Those experiencing severe hypoglycaemia, frequent hypoglycaemia, or impaired hypoglycaemia awareness
- Those with pre-existing microvascular or macrovascular complications from diabetes
- Those with high levels of diabetes distress
- Diabetes duration

Sensitivity analysis may be performed according to assessment of risk of bias, decade of publication to account for technological advancements not accounted for by technology types and excluding studies that define populations as having ‘brittle diabetes’ regardless of diagnostic criteria. Funnel plots may be utilised to investigate for small study effects as well as publication bias.

## Discussion

This systematic review protocol will utilise rigorous methods and pre-determined eligibility criteria to provide a uniquely comprehensive search for a broad spectrum of clinical and economic outcomes in comparing multiple currently available technologies for managing type 1 diabetes. The search strategy for this review was developed in consultation with content and methodological experts. Furthermore, eligibility criteria, risk of bias assessment, and extraction of data will be independently assessed by a team of two reviewers, with a third senior reviewer available to adjudicate any discrepancies.

Limitations of this review include the reliance on published data which may predispose to publication bias. Funding and time constraints dictate limiting the literature search to English language which may also exclude some relevant studies. The differing time periods and international nature of economic evaluations may also limit generalisability and transferability.

To our knowledge, this will be the broadest review of different technologies in type 1 diabetes management. The review will provide insight into the clinical effectiveness of various available technologies and currently lacking data to guide device selection for patients. The inclusion of a variety of technologies also provides the unique opportunity to consider their economic impact with a particular focus on the Australian healthcare system. If adequate evidence is available, this review may provide requisite data for decision modelling as well as a direction for future economic evaluations aimed at health technology for diabetes in Australia.

## Additional files

Additional file 1: Search terms incorporating RCT and economics filters. Search terms were designed for MEDLINE® with daily update and MEDLINE® in-process and other non-indexed citations (via OvidSP). Modifications to search filters for ‘trials’ [35] and ‘economics’ [36] were made. (DOCX 17 kb)

Additional file 2: Populated PRISMA-P checklist [16]. (DOCX 29 kb)

## Abbreviations

AUD: Australian dollars
BGL: Blood glucose level
CGMs: Continuous glucose monitors
CHEERS: Consolidated Health Economic Evaluation Reporting Standards
CI: Confidence interval
CMI: Charlson comorbidity index
CSII: Continuous subcutaneous insulin infusion
CVA: Cerebrovascular accident
DKA: Diabetic ketoacidosis
ESKD: End-stage kidney disease
FGM: Flash glucose monitor
GRADE: Grading of Recommendations, Assessment, Development and Evaluation
HbA1c: Glycated haemoglobin
ICER: Incremental cost-effectiveness ratio
IHD: Ischaemic heart disease
ISPOR: International Society for Pharmacoeconomics and Outcomes Research
MD: Mean differences
MDI: Multiple daily injections
NICE: National Institute for Health and Care Excellence in United Kingdom
PRISMA: Preferred Reporting Items for Systematic Reviews and Meta-Analyses
PRISMA-P: Preferred Reporting Items for Systematic Reviews and Meta-Analysis for Protocols
PVD: Peripheral vascular disease
QALYs: Quality-adjusted life-years
RCT: Randomised controlled trial
RR: Relative risk
SAP: Sensor-augmented pump
SMBG: Self-monitoring of blood glucose (via capillary testing)
USA: United States of America
USD: United States dollars
