# Supplementary material for: Evaluating optimal utilisation of technology in type 1 diabetes mellitus from a clinical and health economic perspective: protocol for a systematic review
Source: Syst Rev. 2018 Mar 12;7:44. doi: 10.1186/s13643-018-0706-9 (PMC5848559; doi:10.1186/s13643-018-0706-9)
Supplement: Supplementary file 1 — Search terms incorporating RCT and economics filters. Search terms were designed for MEDLINE® with daily update and MEDLINE® in-process and other non-indexed citations (via OvidSP). Modifications to search filters for ‘trials’ [35] and ‘economics’ [36] were made. (DOCX 17 kb) [file 13643_2018_706_MOESM1_ESM.docx]

**Search terms with modified filters**

Search terms were designed for MEDLINE® with Daily Update, and MEDLINE® In-Process & Other Non-Indexed Citations (via OvidSP). The search strategy has been translated for other databases.

**CLINICAL SEARCHES:**

1. Diabetes Mellitus, Type 1/
2. (diabet$ adj3 (typ$ 1 or typ$ i or "type1" or "typei" or typ$ one)).ti,ab,ot,hw.
3. (diabet$ adj3 (autoimmun$ or auto immun$ or britt$ or early or juvenil$ or pediatric or paediatric or or sudden onset)).ti,ab,ot,hw.
4. (dm1 or dm 1 or dmt1 or dm t1 or t1dm or t1 dm or t1d or iddm).ti,ab,ot,hw.
5. or/1-4 (89,777)
6. mobile application$ / td,ut
7. mhealth
8. mobile phone$ or tablet$ or connected device$ or wearable$ or smart watch$. ti,ab,ot,hw
9. smartphone / td, ut
10. cell phones / ut
11. or/6-10 (56,487)
12. blood glucose self-monitoring / is,mt,td
13. (blood glucose) adj3 (capillary or finger$).ti,ab,ot,hw.
14. (finger stick) adj3 (device$ or glucose or monitor$).ti,ab,ot,hw.
15. glucometer or glucose meter.ti,ab,ot,hw.
16. or/12-15 (4,277)
17. (sensor$ adj3 (augment$ or pump$)).ti,ab,hw,ot.
18. SAP or SAPT.ti,ab,ot,hw.
19. (minimed or paradigmveo or 6#0g).ti,ab,ot,hw.
20. (paradigm$ adj3 (veo or pump$)).ti,ab,hw,ot.
21. (veo adj3 pump$).ti,ab,ot,hw.
22. ((animas or vibe) adj3 (pump$ or infus$ or system$)).ti,ab,ot,hw.
23. (g4 adj3 platinum).ti,ab,ot,hw.
24. dexcom.ti,ab,ot,hw.
25. or/17-24 (10,336)
26. Insulin Infusion Systems/
27. Pancreas, Artificial/
28. (insulin$ adj3 (pump$ or infus$ or deliver$ or catheter$)).ti,ab,ot,hw.
29. (pump$ adj2 (therap$ or treatment$)).ti,ab,ot,hw.
30. ((subcutaneous adj2 insulin$) or CSII).ti,ab,ot,hw.
31. (artificial adj3 (pancreas or beta cell$)).ti,ab,ot,hw.
32. (closed loop adj3 (hybrid$ or pump$ or deliver$ or infus$ or therap$ or treatment$ or system$)).ti,ab,ot,hw.
33. (accu-chek or cellnovo or dana diabecare or omnipod or omnipod horizon or minimed 670g or smartguard or tandem t*slim).ti,ab,ot,hw.
34. ((integrat$ or dual or combined or unified) adj3 (system$ or device$)).ti,ab,ot,hw.
35. or/26-34 (57,364)
36. Flash adj3 (glucose monitor$ system$ or monitor$).ti,ab,ot,hw.
37. Abbott adj3 (freestyle or libre or flash glucose monitor$).ti,ab,ot,hw.
38. or/36-37 (117)
39. Continuous glucose monitor$
40. Continuous glucose monitor$ adj3 (system$ or device$)
41. Abbott Freestyle Navigator$
42. (g4 adj3 platinum).ti,ab,ot,hw.
43. (g5 adj3 mobile).ti,ab,ot,hw.
44. (dexcom adj3 STS).ti,ab,ot,hw.
45. ((seven or seven plus) adj3 continuous glucose monitor$).ti,ab,ot,hw.
46. dexcom.ti,ab,ot,hw
47. (guardian real-time or guardian real time or enlite) adj3 (sensor or medtronic).ti,ab,ot,hw.
48. or/39-47 (2,543)
49. automated bolus adj3 (calculator$ or advisor$)
50. (ABC or bolus calculator$). ti,ab,ot,hw
51. accu-chek adj3 (connect or aviva expert). ti,ab,ot,hw
52. freestyle adj3 insulinx
53. insulinx
54. or/49-53 (19,690)
55. Insulin/ and Injections, Subcutaneous/
56. (multiple daily adj3 (inject$ or insulin$ or regime$ or routine$)).ti,ab,ot,hw.
57. (multiple dose adj3 (inject$ or insulin$ or regime$ or routine$)).ti,ab,ot,hw.
58. (multiple adj3 (inject$ or insulin$ or regime$ or routine$)).ti,ab,ot,hw.
59. MDI.ti,ab,hw,ot.
60. (injection adj3 therapy).ti,ab,ot,hw.
61. ((basal$ and bolus) adj3 (injection$ or regime$ or routine$ or system$)).ti,ab,hw,ot.
62. (short acting adj3 insulin).ti,ab,hw,ot.
63. (rapid acting adj3 insulin).ti,ab,hw,ot.
64. (fast acting adj3 insulin).ti,ab,hw,ot
65. ((ultra-fast or ultra fast) adj3 (insulin$ or acting insulin$)).ti,ab,hw,ot
66. or/55-65 (17,390)
67. (randomized controlled trial or randomised controlled trial).pt.
68. controlled clinical trial.pt.
69. (randomized or randomised).ab.
70. placebo.ab.
71. randomly.ab.
72. trial.ab.
73. groups.ab.
74. or/67-73 (2,274,138)
75. exp Animals/ not (exp Animals/ and Humans/)
76. 74 not 75 (1,876,139)
77. 11 or 16 or 25 or 35 or 38 or 48 or 54 or 66 (162,595)
78. 77 and 5 (8,068)
79. 78 and 76 (2,369)

**ECONOMIC SEARCH STRATEGY (COMBINED)**

1. economics/
2. exp “costs and cost analysis”/
3. economics, dental/
4. exp “economics, hospital”/
5. economics, medical/
6. economics, nursing/
7. economics, pharmaceutical/
8. (economic$ or cost or costs or costly or costing or price or prices or pricing or pharmacoeconomic$).ti,ab.
9. (expenditure$ not energy).ti,ab.
10. (value for money).ti,ab.
11. budget$.ti,ab.
12. blood glucose self-monitoring / ec
13. monitoring, physiologic / ec
14. diabetes mellitus, type 1 / ec
15. insulin infusion systems / ec
16. insulin / ec
17. hyperglyc*emia / ec
18. hypoglyc*emia / ec
19. diabetes complications / ec
20. cost-benefit analysis
21. models, economic
22. or/80-100 (677,284)
23. ((energy or oxygen) adj cost).ti,ab.
24. (metabolic adj cost).ti,ab.
25. ((energy or oxygen) adj expenditure).ti,ab.
26. or/102-104 (22,919)
27. 101 not 105 (672,224)
28. letter.pt.
29. editorial.pt.
30. historical article.pt.
31. or/107-109 (1,675,906)
32. 106 not 110 (639,894)
33. 111 and 78 (373)
34. 79 Limit English (2,268)
35. 112 Limit English (341)

**An ‘economics’ filter was used with alterations made by review authors:**

Centre for Reviews and Dissemination. Search strategies: NHS EED EMBASE using OvidSP (economics filter). University of York: Centre for Reviews and Dissemination; 2014. [www.crd.york.ac.uk/crdweb/searchstrategies.asp#nhseedembase](http://www.crd.york.ac.uk/crdweb/searchstrategies.asp#nhseedembase). Accessed 2 May 2017.

**A ‘trial’ filter was used with alterations made by review authors:**

Lefebvre C, Manheimer E, Glanville J. Chapter 6: Searching for Studies. In Higgins JPT, Green S (editors). Cochrane Handbook for Systematic Reviews of Interventions. Version 5.1.0 [Updated March 2011]. The Cochrane Collaboration; 2011. <http://training.cochrane.org/handbook>. Accessed 15 December 2017.
